# Engineering of daidzein 3’-hydroxylase P450 enzyme into catalytically self-sufficient cytochrome P450

**DOI:** 10.1186/1475-2859-11-81

**Published:** 2012-06-14

**Authors:** Kwon-Young Choi, EunOk Jung, Da-Hye Jung, Byeo-Ri An, Bishnu Prasad Pandey, Hyungdon Yun, Changmin Sung, Hyung-Yeon Park, Byung-Gee Kim

**Affiliations:** 1 School of Chemical and Biological Engineering, Institute of Bioengineering, Seoul National University, Seoul, South Korea; 2 School of Biotechnology, Yeungnam University, Gyeongsan, Gyeongbuk, South Korea; 3 Interdisciplinary Program for Bioengineering, Seoul National University, Seoul, 151-744, Republic of Korea; 4 Korea Bio-Hub Center, Bio-MAX Institute, Seoul National University, Seoul, South Korea

**Keywords:** Cytochrome P450 monooxygenases, Daidzein 3’-hydroxylase, Self-sufficient P450, CYP102D1

## Abstract

A cytochrome P450 (CYP) enzyme, 3’-daidzein hydroxylase, CYP105D7 (3’-DH), responsible for daidzein hydroxylation at the 3’-position, was recently reported. CYP105D7 (3’-DH) is a class I type of CYP that requires electrons provided through electron transfer proteins such as ferredoxin and ferredoxin reductase. Presently, we constructed an artificial CYP in order to develop a reaction host for the production of a hydroxylated product. Fusion-mediated construction with the reductase domain from self-sufficient CYP102D1 was done to increase electron transfer efficiency and coupling with the oxidative process. An artificial self-sufficient daidzein hydroxylase (3’-ASDH) displayed distinct spectral properties of both flavoprotein and CYP. The fusion enzyme catalyzed hydroxylation of daidzein more efficiently, with a *k*_cat_/K_m_ value of 16.8 μM^-1^ min^-1^, which was about 24-fold higher than that of the 3’-DH-camA/B reconstituted enzyme. Finally, a recombinant *Streptomyces avermitilis* host for the expression of 3’-ASDH and production of the hydroxylated product was developed. The conversion that was attained (34.6%) was 5.2-fold higher than that of the wild-type.

## Introduction

Cytochrome P450 monooxygenases (CYPs) are a super-family of heme-thiolate containing enzymes that catalyze a variety of chemical reactions in regio/stereo-selective manners [1]. Various types of reactions catalyzed by CYP have been identified; generally, these enzymes act as monooxygenases that catalyze the introduction of an oxygen molecule into the substrate whereby one atom of molecular oxygen is incorporated into the product [2]. Oxidation is one of the most valuable reactions in modern industrial processes involving drug development and bio-fuel generation, and is the most common P450-catalyzed reaction for both endogenous and exogenous compounds [3,4].

Fundamentally, the CYP-catalyzed oxidation reaction requires the sequential input of two electrons and two protons ([P450-RH] + 2 e^-^ + 2 H^+^ + O_2_ → P450 + ROH + H_2_O) in the presence of oxygen to activate a R-H bond, followed by the release of the hydroxylated product, R-OH, and water [5]. CYPs require a tight interaction with auxiliary proteins, electron transferring proteins from a cofactor. The combination and orientation of redox proteins classifies CYPs into several types [6-8]. Among them, especially, the class III cytochrome P450 system consists of a self-sufficient CYP, in which the heme domain is fused with a P450 reductase domain and encoded in a single polypeptide [9,10]. Several self-sufficient P450s belonging to the CYP102A family, as well as CYP102D1, have been identified from the genome sequences of various *Bacillus* species such as CYP102A2 and CYP102A3 from *Bacillus subtilis*, CYP102A5 from *B. cereus* and CYP102A7 from *B. licheniformis*, and recently, CYP102D1 was identified from *Streptomyces avermitilis*[11-15]. Especially, the fusion arrangement as self-sufficient CYPs enhances catalytic efficiency, in terms of the kinetic parameters *k*_*cat*_ and K_m_[16]. For example, CYP102A1 BM3 catalyses fatty acid hydroxylation at rates of up to approximately17,000 min^-1^, which is at least two orders of magnitude faster than observed for other class I type fatty acid hydroxylases [6,17]. The knowledge that self-sufficient P450s exhibit the highest turnover frequency due to the higher possibility of contact between the heme and reductase domains, and the induction of intra-molecular electron transfer, have prompted several attempts to make an artificial self-sufficient system by fusing redox proteins. In one study, the N-terminally modified human P450s CYP2C9, CYP2C19 and CYP3A4 fused to the soluble NADPH-dependent oxidoreductase domain of CYP102A1 were constructed to exploit the advantages of the fused nature of the bacterial CYP102A1 system [18]. Also, P450cam from *Pseudomonas putida* (CYP101A) and P450bzo from an environmental metagenome library (CYP203A) were fused with the reductase domain of self-sufficient P450RhF from *Rhodococcus* sp. NCIMB 9784 to increase catalytic efficiency [19].

**Figure 1:**
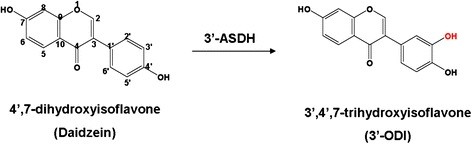
**Chemical structure and oxidative metabolite of daidzein by 3’-ASDH.** This artificial fusion enzyme catalyzes the hydroxylation at the 3’-position of daidzein B-ring with very high regio-selectivity.

Recently our group reported two enzymes, CYP105D7 (3’-DH), which is especially responsible for daidzein hydroxylation at the 3’-position of the daidzein B-ring with very high regio-selectivity, and CYP102D1, a unique self-sufficient P450 from *S. avermitilis*[15,20]. The 3’-DH encoded by *sav7469* from *S. avermitilis* catalyzes the hydroxylation of daidzein to produce 3’,4’,7-trihydroxyisoflavone (3’-ODI) and is a class I CYP, which are necessary electron transfer proteins. CYP102D1 is also a promising model for the construction of an artificial self-sufficient CYP with 3’-DH. Here, we demonstrate the fusion-mediated arrangement of 3’-DH that was achieved by engineering a class I type of CYP into an artificial self-sufficient CYP to increase enzyme catalytic activity and the bio-conversion of daidzein (Figure 1).

Furthermore, P450 systems are especially well-developed in *Streptomyces* strains producing various kinds of secondary metabolites, such as antibiotics that are in most cases regulated by P450 systems at the last step. Many of these systems bestow potent biological activities [21]. Especially, *S. avermitilis* produces 33 P450 enzymes that are valuable as extra- or intra-cellular enzymes capable of catalyzing targeting molecules, as well as more than half of the known biologically active microbial products, including many commercially important antibiotics, immunosuppressive compounds, animal health products and agrochemicals [22]. This vast reservoir of diverse products has made *S. avermitilis* one of the most important industrial microbial genera. A battery of tool for the genetic manipulation of the organism is available [23]. Presently, with the goal of constructing a versatile model host for the biotransformation of daidzein by the heterologous expression of CYP enzymes, the 3’-ASDH gene was amplified with and without 3’-DH gene. The recombinant *S. avermitilis* strains proved to be a suitable natural host for the expression of this artificial fusion enzyme.

## Material and methods

### Chemicals

Daidzein (7,4’-dihydroxyisoflavone) and 3’,4’,7-trihydroxyisoflavone (3’-ODI) used in this study were donated by the Skin Research Institute, Amorepacific R&D Center, South Korea. N,O-bis(trimethylsily)trifluoroacetamide used for derivatization before gas chromatography/mass spectrometry (GC/MS) analyses was obtained from Fluka (Buchs, Switzerland). All other chemicals were of the highest grade available.

### Bacterial strain and culture condition

*S. avermitilis* MA4680 was obtained from the Korea Collection for Type Cultures (KCTC; Daejeon, South Korea). The strain was cultivated on R2YE medium containing 10.3% sucrose, 1% glucose, 1% MgCl_2_ 6H_2_O, 0.025% K_2_SO_4_, 0.5% yeast extract, 0.01% casamino acid, 0.57% TES, 0.005% K_2_HPO_4_, 0.03% CaCl_2_.2H_2_O, 0.003% L-proline, 2 ml of trace element solution and 5 mL of 1 N NaOH. Seed culture of *S. avermitilis* MA4680 was generated in test tubes containing 5 mL R2YE medium by shaking at 220 rpm for 3 days at 28°C. Serial subculture was performed in 50 mL of the aforementioned medium containing 50 μg of thiostrepton for more than 3 days at 28°C.

### Gene manipulation

Chromosomal DNA from *S. avermitilis* MA4680 was prepared using G-spin^TM^ for Bacteria Genomic DNA Extraction kit (iNtRON, Seungnam, South Korea). Plasmids from *Escherichia coli* strains were prepared using the GeneAll DNA Purification System (Geneall Biotechnology, Seoul, South Korea). All polymerase chain reaction (PCR) amplifications by were performed using *Pfu* polymerase with GC II buffer (Takara Bio, Shiga, Japan). Primers used in this study were commercially synthesized by Cosmo Bioscience (Cosmo, Seoul, South Korea). DNA of CYP105D7 and CYP102D1 with codon optimized to suit the codon preference bias of *E. coli* were fully synthesized by Bioneer (Daejeon, South Korea).

To construct the plasmid for the *S. avermitilis* system, Streptomyces expression vector pIBR25 was used. Three sets of over-expressing plasmids (pIBR25::3’-DH, pIBR25::3’-ASDH and pIBR**Δ**3’-DH::3’-ASDH) and one disruption plasmid (pIBR**Δ**3’-DH) were constructed. For each set of primer designs an extra 50 nucleotides harboring the ribosomal binding site region was obtained from the upstream region of the gene. The 3’-DH gene was disrupted in *S. avermitilis* through homologous recombination. To inactivate 3’-DH, the 2.5 kb upstream and 2.5 kb downstream fragments were amplified together with the apramycin resistance gene in the pUC19 vector. Plasmids were digested with restriction enzymes and ligated into the Supercos1 vector. Transformants carrying the single and double crossovers were distinguished by replica plaiting [24]. Finally, four recombinant strains were prepared for the evaluation of daidzein biotransformation by 3’-ASDH.

### Artificial self-sufficient daidzein hydroxylase construction by fusion arrangement and construction of recombinant *S. Avermitilis* expressing 3’-ASDH

The reductase domain of CYP102D1 including the liker domain (VRARQEHERTVFGAADLQTD) was PCR-amplified from a synthetic CYP102D1 gene using a primer set. The PCR reaction conditions consisted of 30 cycles of denaturation for 1 min at 94 °C, annealing for 1 min at 68 °C and extension for 1.5 min at 72 °C. The PCR product was cloned into the pET24ma(+) expression vector (Pasteur Institute, Paris, France) using the restriction enzymes *Sac*I and *Hind*III. The heme domain (from Met1 to Thr402) of CYP105D7 which was chemically synthesized after codon optimization complying with the codon preferences of *E. coli* were digested with *Eco*RI and *Sac*I restriction enzymes (Bioneer, Seoul, Korea). Correctly assembled DNA was cloned into the pET-24ma(+) harboring reductase domain (from Asp478 to Ala1073) of CYP102D1.

To transform the protoplasts *S. avermitilis*, a previously detailed polyethylene glycol (PEG)-associated transformation method was used [25]. The plasmid (1 μg/μL in TE) was added to 0.1 mL of a protoplast suspension containing approximately 10^2^ protoplasts generated by a 15-min lysozyme treatment. Five hundred microliters of the protoplast buffer containing 30% (w/v) PEG1000 (Sigma-Aldrich, St. Louis, MO) was added to the mixture, which was carefully pipetted up-and-down. This protoplast suspension was centrifuged after addition of 300 μL of the protoplast buffer. The suspension was plated into a regeneration medium by gentle shaking. After 16–18 h incubation, the plate was overlaid with 10 μg/mL of thiostrepton.

### Expression and purification of 3’-ASDH and its ultraviolet/visible spectral features

*E. coli* BL21(DE3) cells containing 3’-ASDH was cultured in Luria-Bertani (LB) medium containing 50 μg/mL of kanamycin at 37 °C until the cell concentration reached an optical density at 600 nm (OD_600_) of 0.6, isopropyl-thio-*β*-D-galactopyranoside (IPTG) and δ-aminolevlunic acid as heme precursor were added to a final concentration of 0.25 mM, followed by growing for overnight at 30 °C. Bacteria were harvested by centrifugation and washed twice with phosphate buffered saline (PBS), resuspended in 5 mL of a sonication buffer (pH 7.0) and disrupted by sonication. Disrupted soluble fractions were collected by centrifugation and purified using a Ni-NTA his-tag purification kit (QIAGEN Korea Ltd, Seoul, South Korea). Purification was carried out utilizing the Ni-NTA resin. The Ni-NTA bound enzymes were washed twice with 50 mM potassium phosphate buffer (pH 7.0) containing 500 mM NaCl and 50 mM imidazole, and enzymes were eluted with the same buffer containing 200 mM imidazole. Finally, imidazole and sodium chloride were removed by dialysis against 50 mM potassium phosphate buffer (pH 7.0). Purified proteins were subjected to 10% sodium dodecyl sulfate-polyacrylamide gel electrophoresis (SDS-PAGE) and spectrophotometric analysis to measure carbon monoxide (CO)-binding activity [26]. Absorption spectra of CO-bound recombinant CYP proteins after sodium dithionite reduction were measured by UV/vis spectrometry (Thermo Lab Systems, Beverly, MA, USA) by scanning wavelength from 400–500 nm [26]. The protein concentration was estimated using reduced CO versus reduced difference spectra. The 3’-ASDH protein content was determined using an extinction coefficient of 91.9 mM^-1^ cm^-1^ at 450 nm [26].

### Determination of kinetic values of 3’-ASDH

NADPH oxidation was measured by the absorption decrease at 340 nm with UV/vis spectrometry (Thermo Lab Systems, Beverly, MA, USA). A solution containing 160 μL of potassium phosphate buffer (pH 7.5), 10 μL of each substrate concentration solution in dimethyl sulfoxide (DMSO) and 20 μL of enzyme solution (10 μmol/mL) was incubated for 5 min in a 96-well microplate. The reaction was started by adding 10 μL of a 10 mM NADPH solution. For determination of kinetic parameters, a substrate concentration range of 2.5–50 μM of daidzein was used. All data were fitted to the Michaelis-Menten equation by linear regression. As a reference sample, NADPH consumption rates were measured in the absence of substrate.

### Substrate binding spectra of each CYP (K_d_ value)

Spectroscopic substrate binding spectra were obtained as described previously [17]. A solution of substrate was added to the enzyme solution to produce a final substrate concentration of 2.5–100 μM. For the determination of K_d_ values, the reciprocal values of the absorption difference between 394 nm and 418 nm were plotted against the reciprocal values of the substrate concentration. For calculation of the K_d_ value of the enzyme-substrate, the experimental data were fitted to a hyperbola curve (y = ax(b + x)^-1^) by a nonlinear regression procedure based on the Durbin-Watson algorithm in SigmaPlot software (Systat, San Diego, CA).

### Resting cell assay of *E. Coli* BL21(DE3) expressing 3’-ASDH in the bioconversion of daidzein

Cells were harvested and washed twice with 50 mL of PBS buffer. And the cells were resuspended in potassium phosphate buffer (100 mM, pH 7.0) containing 1% of glucose. The initial substrate stock solution was prepared by dissolving daidzein (24.5 mg) into the DMSO and diluted to reaction concentration (100 μM) using DMSO: MeOH (50:50) solvent. Whole cell reactions were initiated by the addition of the substrate at 37 °C in a shaker (200 rpm) for 24 h. The reaction was quenched by the addition of same volume of ethyl acetate to the reaction, followed by vigorous vortexing. The mixtures were centrifuged at 13,000 rpm for 10 min, after which the upper layer containing any remaining substrate and products (oraganic layer) was extracted, followed by evaporation of the supernatant using a vacuum (BioTron, Bucheon, South Korea). The sample was subsequently used for GC/MS structural and quantitative analyses.

### Resting cell assay of recombinant *S. Avermitilis* in the bioconversion of daidzein

Each recombinant *S. avermitilis* cells harvested from 50 mL of culture broth were washed twice with potassium phosphate buffer (50 mM, pH 7.2). After centrifugation (13,000 rpm, 10 min), 5 g of cells (wet wt.) were added to 50 mL of potassium phosphate buffer (100 mM, pH 7.2) with 0.1 mM of final substrate concentration in DMSO:MeOH (50:50). The mixture containing 50 mL in 250 mL flask was incubated in a shaker at 200 rpm for 72 h at 28 °C and the reaction mixture was extracted with ethylacetate (JUNSEI, Japan). The extracted sample was evaporated in a centrifugal vacuum concentrator (BioTron, South Korea). And the solvents in DMSO:MeOH (50:50) was added in the reaction with the final volumetric ratio from 0% to 40% in order to examine the biotransformation conversion depending on organic solvent concentrations.

### GC/MS analysis of products

For structural analysis of oxidative metabolites of daidzein, samples extracted with ethylacetate were evaporated to dryness and dissolved in 100 μL methanol. The same volume of 0.5 M NH_4_CO_3_ and 2 M KCl was added to each sample, and the mixture was centrifuged at room temperature, after which the supernatant was transferred to a new vial for derivatization. To protect hydroxyl groups, reaction products were derivatized using N,O-bis(trimethylsilyl)trifluoroacetamide (BSTFA) by heating at 60 °C for 60 min. GC/MS was performed using a Finnigan MAT system (Gas chromatograph model GCQ, HP 19091j-433; Thermo Labsystems, USA) connected to an ion trap mass detector. The BSTFA derivatives were separated through a non-polar capillary column (5% phenyl methyl siloxane capillary 30 m × 250 nm i.d., 0.24 μm film thickness, HP-5) containing a linear temperature gradient (for fatty acids: at 100 °C 0.5 min, 5 °C/min to 200 °C, hold for 5 min, 10 °C/min to 250 °C, hold for 3 min; for cyclic compounds: at 60 °C 1 min, 30 °C/min to 250 °C, hold for 10 min, 1 °C/min to 275 °C, hold for 3 min). The injector port temperature was 250 °C. Mass spectra were obtained by electron impact ionization at 70 eV, and scan spectra were obtained within the range of 100–600 m/z. Selected ion mode (SIM) was used for the detection and fragmentation analysis of major products.

### Homology modeling of 3’-ASDH

Homology modeling of 3’-ASDH was performed by combinations of heme domain of CYP105D7 and reductase domain of CYP102D1. The homology model of CYP105D7 was constructed using 3ABB [27], a crystal structure of CYP105D6 involved in filipin hydroxylation, and CYP102D1 was constructed using 3hf2a, a crystal structure of CYP102A1 variant from the PDB database as a template. The predicted 3D structure was constructed based on a comparative homology modeling method using the Genefold and Composer programs from the SYBYL software package (Tripos Associates, St. Louis, MO). The initial state model was energy-minimized by the conjugate gradient method at an energy gradient norm of 0.01 kcal/mol.

## Results

### Fusion-mediated self-sufficient construction of CYP105D7 with the reductase domain of CYP102D1

**Figure 2:**
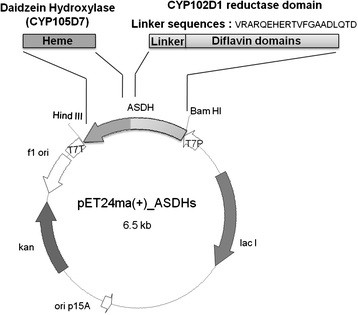
**Design of artificial self-sufficient daidzein hydroxylases by heme domain swapping with self-sufficient CYP102D1 from*Streptomyces avermitilis*.** The reductase domain consisting of di-flavin binding domain (FAD/FMN) has linker sequences from Val458 and the coding synthetic DNA was first cloned into the expression vector pET24m(a). And the target DNAs coding daidzein hydroxylases were amplified with each specific primer and coned into the pET24m(a)-CYP102D1 reductase.

A recent study described the functional expression of a self-sufficient P450, CYP102D1, from *S. avermitilis* in *E. coli*[15]. Since the original gene coding CYP102D1 could not be successfully expressed in *E. coli*, the gene was chemically synthesized following codon optimization complying with the codon preferences of *E. coli*. The reductase domain consisting of the di-flavin binding domain (FAD/FMN) has linker sequences from Val458 and the coding synthetic DNA was first cloned into the expression vector pET24m(a). Target DNAs encoding daidzein hydroxylases were amplified with each specific primer and cloned into the pET24m(a)-CYP102D1 reductase (Figure 2). Especially, the *sav7469* gene coding 3’-DH was not solely expressed in *E. coli* well; the codon optimized *sav7469* gene missing stop codon based on *E. coli* codon bias was also used in the artificial self-sufficient P450 construction. Finally, the 3’-ASDH fusion protein was prepared by two synthetic DNA gene manipulations (Figure 2).

### Expression of artificial fusion proteins and their spectroscopic characterizations

**Figure 3:**
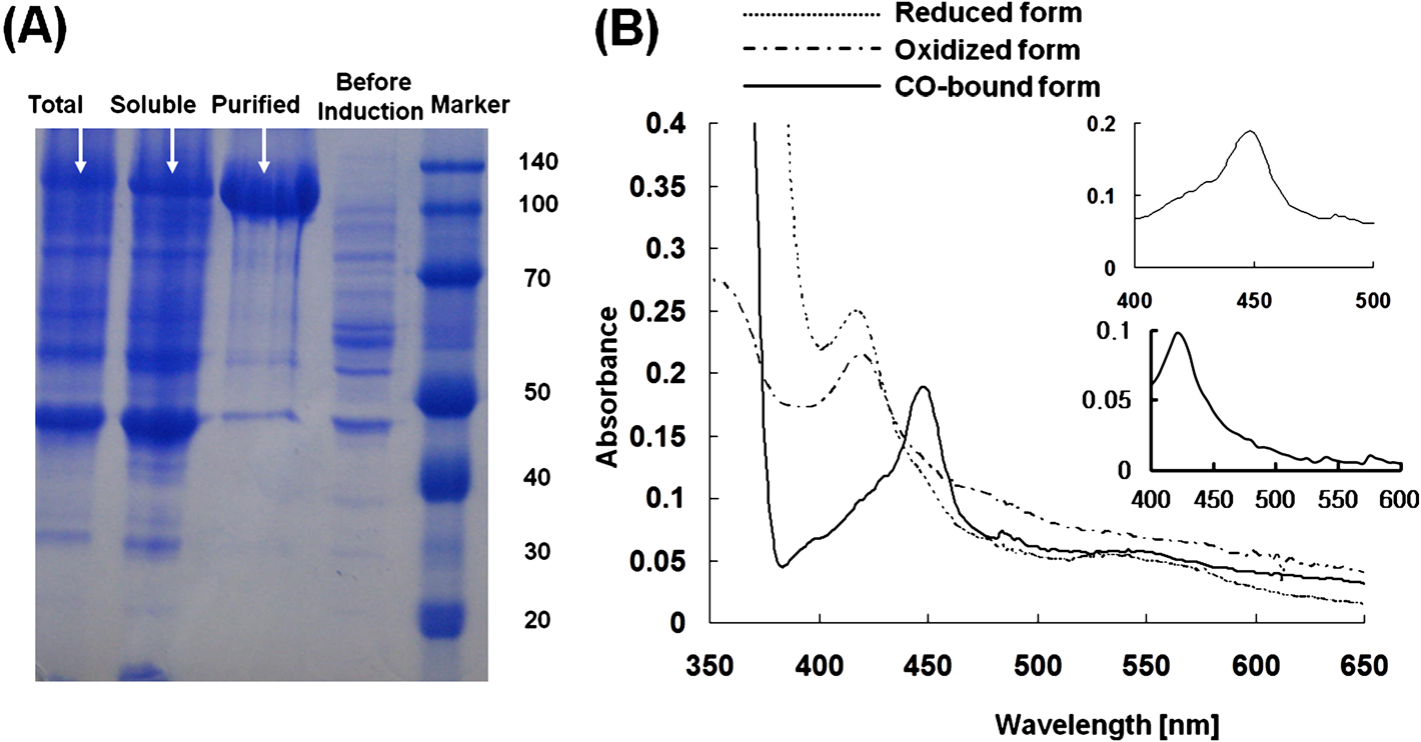
**Expression and UV-visible absorption spectra of 3’-ASDH.** (**A**) SDS/PAGE analysis of total cell protein content as well as purified protein containing the His-6 tag; the lanes, from the left, are total cell protein content after induction, total soluble protein content, purified protein content and total cell protein content before IPTG and protein marker, respectively. (**B**) UV-visible absorbance spectra of purified 3’-ASDH fusion protein revealed spectral features characteristic of CYPs; Dashed line: oxidized form, Dotted line: Reduced form with dithionite. Solid line: CO-difference spectra; Inserted figure: CO-difference spectra of 3’-ASDH.

Figure 3A summarizes the expression of the constructed fusion protein. The protein was abundantly expressed as a soluble fraction yielding 19.2 mg/L of 3’-ASDH, and His-tag purified protein was prepared for *in vitro* reaction as a reconstituted system. The purified fusion protein displayed a single protein band corresponding to a theoretical molecular weight of about 110 kDa, which was deduced by taking the reductase domain of CYP102D1 as 65 kDa, and finally concentrated (×1500) to yield approximately 3.0 mg/mL (1.0 μM) (Figure 3A).

To examine the functional folding of the purified protein in the ferric state (reduced form), UV/vis spectroscopy was utilized. In general, a cysteine-thiolate ligand coordinated to the heme iron atom of CYP regulates any spectroscopic and catalytic properties. Figure 3B shows the absorption spectra of the fusion protein. Absorption spectroscopy of the purified artificial self-sufficient protein in its oxidized form (dashed line) and reduced form (dash-dotted line) revealed general features of bacterial CYPs. The oxidized form of spectra shows typical spectra of self-sufficient CYP enzyme with Soret peak at 419 nm, an α-band at 567 nm, and and unresolved β-band due to the overlap with a broad shoulder in the 440–490 nm regison. This shoulder was interpreted as the flavin prosthetic groups. The 440–490 nm shoulder disappears upon addition of sodium dithionite owing to reduction of the flavins [28]. In addition, the absorption spectra of the reduced-CO oxidized forms are indicated in the inset. The CO-difference spectra resulting from the insertion of CO gas into a reduced state by sodium dithionite revealed that the heme Soret absorption maximum was at 450 nm [19]. As the reductase domain of CYP102D1 uses NADPH as an electron donating source, the constructed fusion enzyme used NADPH preferably (approximately 500 μmol cyt *c*/min/μmol CYP).

### Daidzein hydroxylation reaction catalyzed by 3’-ASDH fusion enzyme

The activity of 3’-ASDH fusion enzyme was measured using daidzein as a substrate in order to validate the efficiencies of fusion orientations with the natural three-component system. Previously, the CamA/B redox proteins from *Pseudomonas putida* were proven to be alternative electron transferring proteins [20]. Thus, the reconstituted 3’-DH and CamA/B enzyme activity was compared to that of 3’-ASDH. GC-MS assay of daidzein hydroxylation activity measured the hydroxylated products. The kinetic parameters of the 3’-ASDH enzyme were incredibly dominant over those of the reconstituted 3’-DH-camA/B system (Table 1), indicating that the fusion arrangement in a single polypeptide was absolutely efficient for inter-molecular electron transfer, rather than intra-molecular electron transfers, and was sufficient for CYP activity. As mentioned before, an excellent benefit of the fusion arrangement is that the covalent linkage presumably stabilizes the interaction between P450 and redox protein, enhancing electron transfer efficiency. As such, one would expect this to improve the catalytic activity in terms of *k*_*cat*_, whereas the substrate specificity would not be changed significantly. The experimental results supported these expectations; the *K*_*m*_ values of the reconstituted 3’-DH and 3’-ASDH proteins were quite similar in the range of approximately 20 μM, whereas the catalytic activity (*k*_*cat*_) of the fused enzymes was significantly different from that of 3’-DH alone. The *k*_*cat*_ values of 3’-ASDHs were approximately 342 nmol/min-^1^/nmol of CYP, which is about 24-fold higher than that of the reconstituted 3’-DH-camA/B system. These results indicate that the binding affinity (K_d_ =15.3 μM) between the heme domain of the DH enzyme and the substrate daidzein was not affected by fusion arrangement, whereas artificial fusion arrangements allowed effective intra-molecular electron transfer from NADPH to the heme domain through reductase, confirming our expectations. Effective electron transfer between CYP and reductase existing in different molecules of a fusion monooxygenase can occur if the two molecules are dimeric partners, but this type of electron transfer cannot be discriminated from intramolecular transfer if the dissociation constant of the dimer is very small [15]. Such intramolecular electron transfer has been reported for naturally-occurring bacterial fusion CYP enzymes such as CYP102A family members catalyzing fatty acid hydroxylation.

**Table 1:** Comparison of kinetic parameters of P450s with natural redox partners and P450s fused to aself-sufficient mimic system

**Kinetic parameters**	**3’-DH-CamA/B**	**3’-ASDH**
*K*_*m*_ [*μM*]	21.8 ± 3.6	20.5 ± 5.1
*k*_*cat*_	15.0 ± 2.1	342.3 ± 53.6
[*min*^*-1*^]		
*k*_*cat*_*/K*_m_	0.7 ± 0.4	16.8 ± 4.6
[*μM*^*-1*^ *min*^*-1*^]		
Coupling efficiency (%)	< 55	< 72

### Quantitative and structural analysis of reaction products using high-performance liquid chromatography (HPLC) and GC/MS

**Figure 4:**
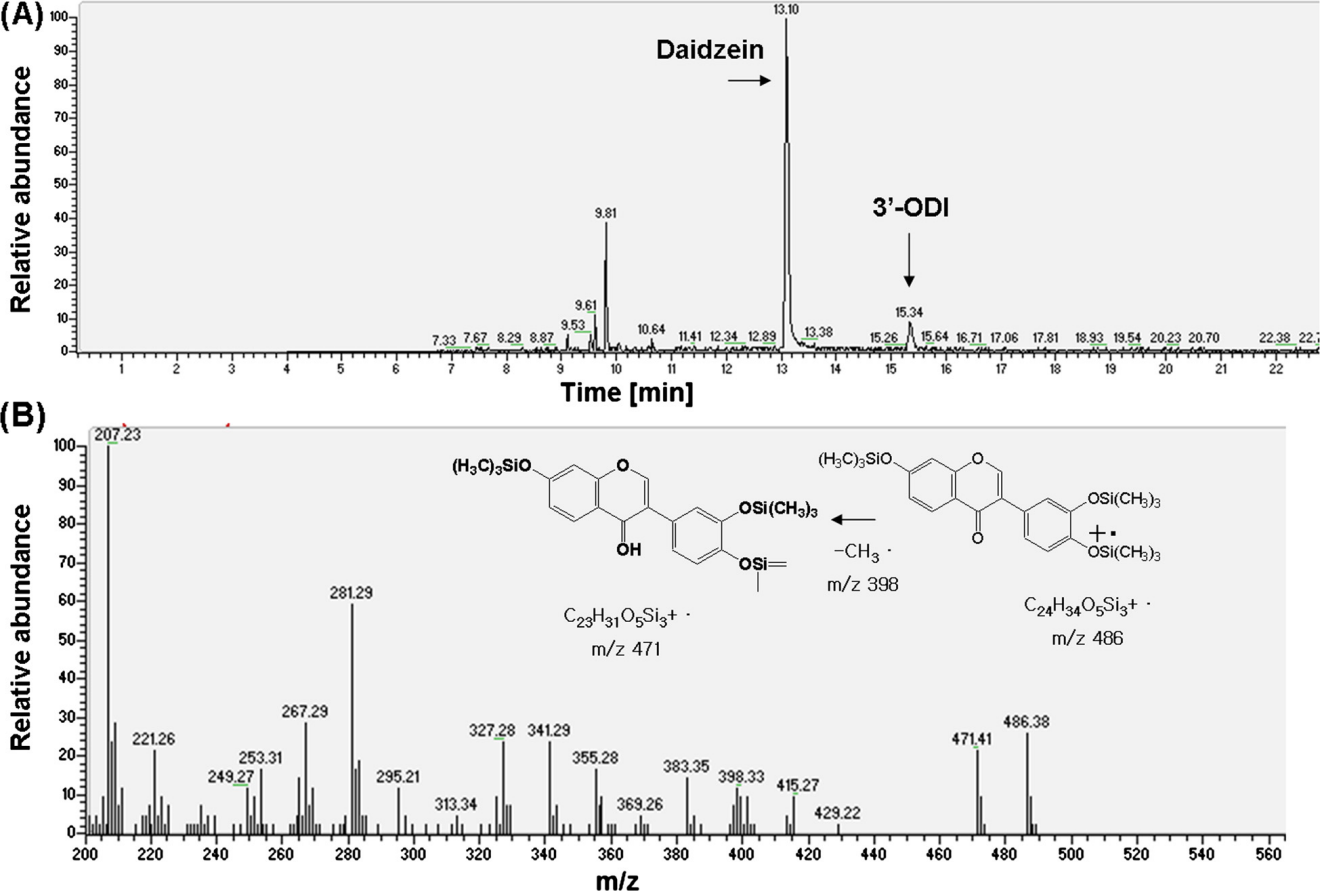
**(A) Gas-chromatography analysis patterns of monohydroxylated product**. A major oxidative metabolite of daidzein was separated and identified as 3’,4’,7-trihydroxyisoflavone (3’-ODI) based on the retention times and mass (m/z) values comparing with reference compound. (**B**) Mass spectral analysis revealed that each had a molecular ion mass of 486 m/z after BSTFA derivatization, which was exactly the same as those of the reference compounds. As a result, 3’-ASDH oxidized daidzein to mono-hydroxylated product of daidzein with very high catalytic activity.

The resting cell assay using 3’-ASDH expressing *E. coli* BL21(DE3) revealed unique reaction products. The identification of oxidative metabolite was performed with GC/MS^n^ (Figure 4A). Mass analysis of daidzein revealed a molecular ion mass of 398 m/z, and each monohydroxylated product was observed at 486 m/z, resulting in a 88 m/z increase due to incorporation of molecular oxygen (+16 m/z) after BSTFA derivatization (+72 m/z) (Figure 4B). These values agree with the mass analysis patterns of the corresponding reference chemicals. The hydroxylated products were identified as 3’-ODI, which was mono-hydroxylated form of daidzein at the *ortho* position of 4’-hydroxyl group at the B-ring. Mass spectral analysis revealed that each had a molecular ion mass of 486 m/z after BSTFA derivatization, which was exactly the same as those of the reference compounds. This result demonstrates that our previous understanding of daidzein hydroxylase reserves regio-selectivity after fusion arrangement with reductase domain regardless of catalytic activity for aromatic compounds such as ortho-hydroxylated daidzein.

### Development of recombinant *S .Avermitilis* host system for heterologous expression of 3’-ASDH

The resting cell assay in *E. coli* revealed that 3’-ASDH expressed in *E. coli* did not produce the expected conversion (approximately 5%) when 100 μM of daidzein was used as an initial substrate concentration, although purified 3’-ASDH enzyme showed very high catalytic activity.

Previously we reported that *S. avermitilis* strain is highly active and regiospecific to daidzein and genistein, producing monohydroxylated products [20,29,30]. The *sav7469* gene encoding 3’-DH, among the 33 CYPs of *S. avermitilis*, dominantly catalyzed the regio-specific hydroxylation of daidzein to produce 3’-ODI. These results suggested that *S. avermitilis* is a very good reaction host for daidzein biotransformation by virtue of having the genomic encoding capacity for 3’-DH and its natural redox proteins. Thus, we presently sought the heterogeneous expression of 3’-ASDH in *S. avermitilis* and biotransformation of daidzein using recombinant strains.

Four recombinant *S. avermitilis* strains were constructed to compare the productivity of 3’-ODI, *S. avermitilis*Δ3’-DH, *S. avermitilis* Δ3’-DH::3’-ASDH, *S. avermitilis*::3’-DH and *S. avermitilis*:: 3’-ASDH (Table 2). As the *S. avermitilis* wild-type genome encodes 3’-DH (*sav7469*), the wild-type alone could convert daidzein into 3’-ODI at a rate of 6.7%. After deletion of the *sav7469* gene, almost 50% of the wild-type activity was retained, whereas strain Δ3’-DH::3’-ASDH restored conversion and the conversion yield was 2.3-fold higher than that of the wild-type, suggesting that 3’-DH is responsible for daidzein hydroxylation at the 3’-position. Further investigation of the productivity by *S. avermitilis* Δ3’-DH::3’-ASDH strain revealed that 3’-ASDH was functionally expressed in *S. avermitilis* and could produce 3’-ODI with a conversion rate of 15.5% without any background influence by 3’-DH in its genome. These results indicated that artificial self-sufficient fusion enzymes could work in the *S. avermitilis* recombinant strain, and its catalytic activity in terms of product production was increased by more than 4 times along with enzyme kinetic parameters.

**Table 2:** **Comparison of conversions of recombinant*E. coli*and*S. avermitilis*for 3’-ODI production**

**Strains**	**Conversion**	**Productivity**	**Fold-change**
	(%)*	**(*μg/hr/g cells)***	
*E. coli* BL21::ASDH	2.1	2.9	0.3
Wild type *S. avermitilis*	6.7	9.3	1.0
Δ3’-DH	3.4	4.7	0.5
Δ3’-DH::3’-ASDH	15.5	21.4	2.3
Wild type::3’-DH	22.0	30.5	3.3
Wild type:: 3’-ASDH	34.6	48.4	5.2

*100 μM of substrate was used as an initial concentration at each reaction.

### Computational structure prediction of 3’-ASDH and verification of electron transfer distance

In this study, daidzein was hydroxylated by oxidation catalyzed by the fusion enzyme, wherein an additional diflavin containing the reductase responsible for transfer of a reducing equivalent from NADPH to CYP heme was additionally fused including original linker sequences. Since this orientation of the electron transfer system was not native, the distance between the heme iron and flavin was very important regarding enzyme activation and coupling efficiency. Here, we used an original linker domain in the sequence of CYP102D1, so as not to change its natural folding or the flexibility of linker peptides (VRARQEHERTVFGAADLQTD). To verify electron transfer, we generated computational models of both native CYP102D1 and 3’-ASDH, and measured the heme-FMN distances.

**Figure 5:**
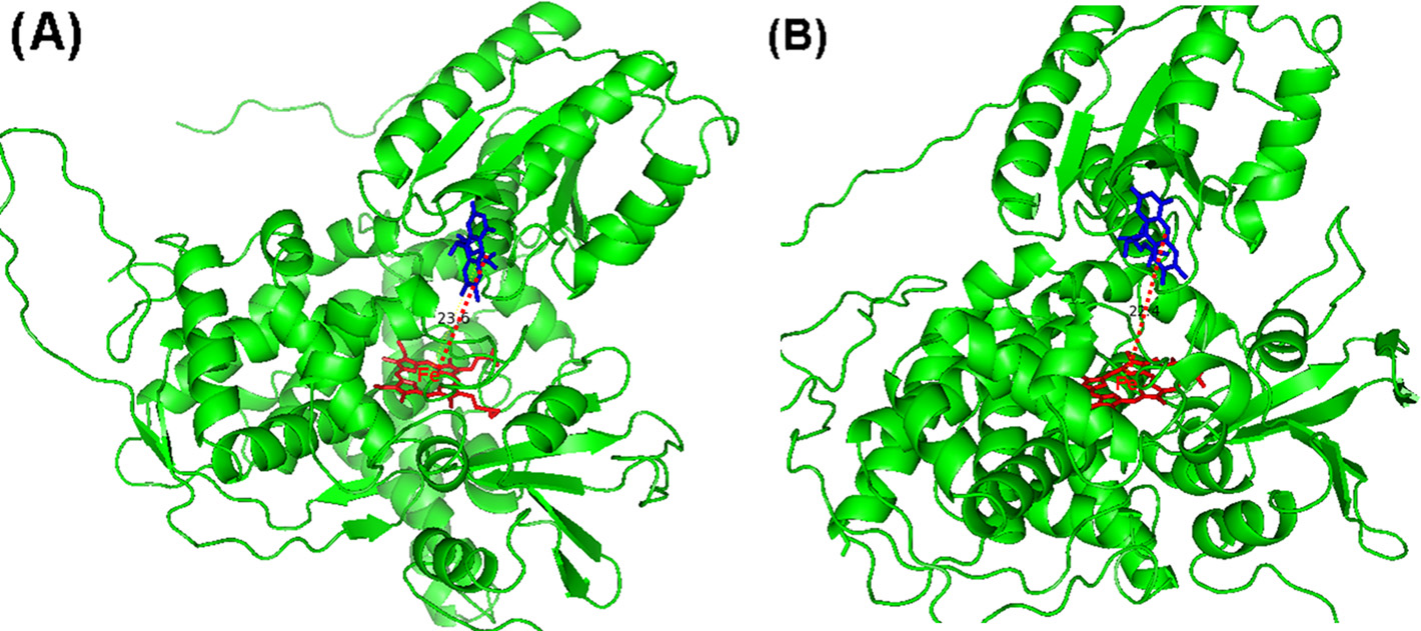
**Computational simulation revealing coincident incorporation of heme and flavin structures in the protein.** Here, the flavin structure was located close to unnative heme iron center within a calculated distance of 22.4 Å (Figure 5B), which is very reasonable for the inter-molecular transfer of electrons based on the distance of 23.6 Å (Figure 5A) between FMN and heme center in the native CYP102D1 enzyme structure.

Computational simulation revealed that the ASDH enzyme had a modular structure in which FAD/FMN and NADPH binding sites were situated distinctively at the N-terminal, central and C-terminal regions. As shown in Figure 5, both the heme and flavin structures were incorporated together in the protein. The flavin structure was located close to the non-native heme iron center within a calculated distance of 22.4 Å (Figure 5B), which was very reasonable for the inter-molecular transfer of electrons based on the distance of 23.6 Å (Figure 5A) between FMN and heme center in the native CYP102D1 enzyme structure [17]. Although the conformational changes induced by interaction between reductase and CYP or substrate were not exactly predicted or calculated, sufficient electron transfer took place and enzymatic reactions occurred very quickly. Further investigations of this CYP-flavoprotein system, its electron transfer rate, and the coupling efficiency between the reconstituted and fused systems are underway.

## Discussion

In synthetic chemistry, many synthetic strategies for carbon-hydrogen (C-H) oxidation rely on a reactive intermediate that involves subtle differences in C-H bond strength to achieve regio-selectivity. Owing to the large number of C-H bonds in most bioactive chemicals, identifying a reagent that can specifically react at one C-H bond is usually difficult. Thus, concerning regio-selectivity, enzymatic oxidation catalyzed by P450s on non-activated carbon is a great challenge in modern industrial processes such as drug development and bio-fuel generation.

As a model system, we demonstrated the regio-specific hydroxylation of daidzein of which their biological activities or functions were greatly affected to obtain more than 30 times higher anti-oxidant capacity by hydroxylation on its 3’-position using an artificial self-sufficient enzyme [31,32]. Here, we solved the requirement for redox proteins, which is considered to be a bottle neck in CYP enzyme reaction, by fusion construction. As the synthetic 3’-ASDH gene was successfully expressed in *E. coli* after the codon optimization process, we were able to compare its catalytic activity with that of the 3’-DH-CamAB system [25]. The fusion-enabled enzyme displayed a higher turn-over rate and higher conversion without any alterations of regio-specificity and substrate specificity, compared with the parent enzyme.

Use of *E. coli* as a biotransformation system carries many limitations. For example, the bacteria require exogenous transfer of electron from redox proteins such as CamA and CamB [30]. In addition, *E. coli* are very susceptible to the killing action of organic solvents used in dissolving substrate in the reaction [33,34]. As an alternative organism, *Streptomyces* preserves high redox potential during enzymatic reactions; the organism has proved useful for production of various antibiotics or secondary metabolites, which need many redox proteins. As well, *Streptomyces* is less toxically affected by organic solvents [35]. These attributes have been exploited in the use of *Streptomyces* as a CYP reaction host for daidzein biotransformation. Furthermore, it is possible to dissolve substrate in the reaction up to more than final volume of 1 mM when using 10% of DMSO:MeOH (50:50) mixed solvents without any inhibition of cell growth or cell toxicity (Table 3) [29]. With these characteristics in combination with gene manipulation systems like over-expression and gene disruption, *S. avermitilis* could be a strong host for biotransformation of daidzein [29].

**Table 3:** **Comparison of conversions of recombinant*Wild type:: 3’-ASDH*for 3’-ODI production depending on the concentrations of DMSO**

**DMSO:MeOH**	**0**	**5**	**10**	**20**	**40**
**(50:50) (%)**					
Conversion (%)*	20.5	26.7	34.6	4.5	n.d.

*100 μM of substrate was used as an initial concentration at each reaction.

n.d. not determined.

Although *S. avermitilis* has been used for the heterologous expression or secretion of several polypeptides of bacterial and eukaryotic origin, heterologous genes usually need genetic modification in ribosomal binding sites or in its full DNA sequences for successful expression. However, 3’-DH and reductase domain originate from *S. avermitilis* and no genetic modifications was necessary for expression. Disruption of sav7469 (3’-DH) regarded as a major factor in 3’-ODI production and overexpression of 3’-ASDH in this deletion mutant finally proved that it is well-expressed in its original host and operates in daidzein biotransformation more efficiently as well. Although only 3’-DH was not operative in daidzein hydroxylation, considering that *S. avermitilis*Δ3’-DH retained some activity for 3’-ODI production, the conversion of daidzein using *S. avermitilis*::3’-ASDH strain increased more than 4-times. Compared to the conversion rate of <5% in *E. coli* biotransformation, this is an absolutely increased value. This yield is very close to the levels that are attractive for industrial applications.

This study regarding the combination of enzyme engineering and *S. avermitilis* host development opens up the possibility of actinomycetes utilization as a recombinant expression host system for the production of biologically important compounds using fusion P450 proteins.

## Competing interests

The authors declare that they have no competing interests.

## Authors’ contributions

CKY, JDH, ABR, SCM and PBP carried out the molecular genetic studies and structural analysis of samples. And JE and PHY participated in the sequence alignment and computer simulations. Finally YHD and KBG drafted and revised the full manuscript. All authors read and approved the final manuscript.
